# ASCs-Exosomes Recover Coupling Efficiency and Mitochondrial Membrane Potential in an *in vitro* Model of ALS

**DOI:** 10.3389/fnins.2019.01070

**Published:** 2019-10-17

**Authors:** Elisa Calabria, Ilaria Scambi, Roberta Bonafede, Lorenzo Schiaffino, Daniele Peroni, Valentina Potrich, Carlo Capelli, Federico Schena, Raffaella Mariotti

**Affiliations:** ^1^Department of Neurosciences, Biomedicine and Movement Sciences, University of Verona, Verona, Italy; ^2^Department of Translational Genomics, Centre for Integrative Biology, University of Trento, Trento, Italy; ^3^Department of Physical Perfomances, Norwegian School of Sport Sciences, Oslo, Norway

**Keywords:** NSC-34 cell line, mitochondria, ALS, high resolution respirometry, membrane potential, exosomes, complex I, coupling efficiency

## Abstract

The amyotrophic lateral sclerosis (ALS) is a fatal neurodegenerative disorder characterized by motoneurons death. Mutations in the superoxide dismutase 1 (SOD1) protein have been identified to be related to the disease. Beyond the different altered pathways, the mitochondrial dysfunction is one of the major features that leads to the selective death of motoneurons in ALS. The NSC-34 cell line, overexpressing human SOD1(G93A) mutant protein [NSC-34(G93A)], is considered an optimal *in vitro* model to study ALS. Here we investigated the energy metabolism in NSC-34(G93A) cells and in particular the effect of the mutated SOD1(G93A) protein on the mitochondrial respiratory capacity (complexes I-IV) by high resolution respirometry (HRR) and cytofluorimetry. We demonstrated that NSC-34(G93A) cells show a reduced mitochondrial oxidative capacity. In particular, we found significant impairment of the complex I-linked oxidative phosphorylation, reduced efficiency of the electron transfer system (ETS) associated with a higher rate of dissipative respiration, and a lower membrane potential. In order to rescue the effect of the mutated SOD1 gene on mitochondria impairment, we evaluated the efficacy of the exosomes, isolated from adipose-derived stem cells, administrated on the NSC-34(G93A) cells. These data show that ASCs-exosomes are able to restore complex I activity, coupling efficiency and mitochondrial membrane potential. Our results improve the knowledge about mitochondrial bioenergetic defects directly associated with the SOD1(G93A) mutation, and prove the efficacy of adipose-derived stem cells exosomes to rescue the function of mitochondria, indicating that these vesicles could represent a valuable approach to target mitochondrial dysfunction in ALS.

## Introduction

Amyotrophic lateral sclerosis (ALS) is a fatal late onset neurodegenerative disease, characterized by selective degeneration of motoneurons. In the 90–95% of cases, the disease is sporadic, while in the 5–10% is familial (Bonafede and Mariotti, 2017). The first mutated gene associated with ALS was Cu/Zn-superoxide dismutase 1 (SOD1), and several point mutations of this gene have been reported, as the G93A. The SOD1 protein is localized in the cytoplasm and in the intermembrane space of mitochondria. The mutations cause a misfolding of the protein, that aggregates and interferes with several molecular pathways, leading to motoneuron degeneration (Wiedau-Pazos et al., 1996; Kruman et al., 1999; Cozzolino and Carrì, 2012). In addition, the misfolded SOD1 protein compromises mitochondrial function and induces morphological changes, such as swelling and formation of vacuoles in the mitochondrial matrix (Raimondi et al., 2006; Martin et al., 2007). Mitochondria are involved in energy production (ATP), cellular respiration, calcium homeostasis and are responsible for the production of reactive oxygen species (ROS). Alterations of mitochondria regarding their ultrastructure, morphology and the dysfunction of elements of the electron transfer system (ETS) have been reported in motoneurons, muscles, post-mortem brain tissue, lymphocytes, and liver of ALS murine models and patients (Nakano et al., 1987; Bowling et al., 1993; Curti et al., 1996; Wiedemann et al., 1998; Vijayvergiya et al., 2005).

The mitochondrial defects associated with ALS have been studied on isolated enzymes or purified mitochondria (Mattiazzi et al., 2002; Menzies et al., 2002). In ALS patient’s lymphocytes, enzymatic assays on NADH-ferricyanide reductase showed a reduction of complex I activity compared to controls (Ghiasi et al., 2012). In patient fibroblasts, the evaluation of mitochondrial function revealed a specific deficit in ALS patients compared to control groups (Walczak et al., 2019). In isolated coupled mitochondria obtained from SOD1(G93A) mice tissues (brain, liver, and spinal cord) at disease onset, a reduced complex I-linked phosphorylative activity (sustained by glutamate and malate) was reported (Mattiazzi et al., 2002). A recent systematic review points out an early and lasting reduction of complex I activity and respiratory rates alterations in transgenic SOD1(G93A) mice, compared to wild-type (Irvin et al., 2015). In primary SOD1(G93A) motoneurons, lower mitochondrial membrane potential and impairment of calcium homeostasis were found compared to controls (Kruman et al., 1999). Specific silencing of the SOD1(G93A) tans-gene significantly delays the onset of the disease and improves the life-span of these mice (Xia et al., 2006). These studies indicated that the mitochondrial alterations found in ALS patients occur also *in vitro* and *in vivo* ALS models, supporting their use to test possible new therapeutic approaches acting on mitochondrial dysfunction.

A novel therapeutic strategy proposed for neurodegenerative disease concerns the use of exosomes derived from stem cells. Exosomes are extracellular vesicles released from all cell types and are able to recapitulate the efficacy of the origin cells. To this purpose, exosomes isolated from stem cells are used as a possible therapy in different neurodegenerative diseases, instead of using the parental cells and avoiding the possible consequences of cell therapy (Bonafede and Mariotti, 2017). We recently reported that exosomes isolated from adipose-derived stem cells (ASCs, ASCs-exosomes) are neuroprotective inhibiting apoptosis in an *in vitro* ALS model, the motoneuron-like cell line (NSC-34) (Bonafede et al., 2016). Since mitochondria are involved in the cellular apoptotic pathways through the release of cytochrome c, their dysfunction may exacerbate the susceptibility and death of motoneurons in ALS (Kruman et al., 1999). Moreover, it has been reported that the treatment of primary neuronal cells with ASCs-exosomes, alleviate the aggregation of SOD1 mutated protein and normalize the phospho-CREB/CREB ratio and PGC-1α expression level (Lee et al., 2016). However, the specific action of ASCs-exosomes on mitochondrial respiratory pathways remains to be clarified.

In this study, we used the murine NSC-34 cell line since they express the typical physiological and morphological properties of motoneurons. Moreover, to mimic the motoneuron phenotype in ALS, they were stably transfected with the human mutant SOD1(G93A) gene (Bonafede et al., 2016). We investigated the alterations of mitochondrial function concerning the relative contributions of mitochondrial complexes and the coupling efficiency in the *in vitro* model of ALS. To this purpose, we used the high resolution respirometry (HRR), a technique that allows studying mitochondrial respiratory capacity (complexes I-IV), integrity and energy metabolism in intact or permeabilized cells. The intact cells were analyzed in cell culture media, ensuring availability of substrates and appropriate ionic composition to maintain the cell membrane potential and intact signaling. In this condition, mitochondrial activity is related to the use of endogenous substrates (Pesta and Gnaiger, 2012). On the other hand, the use of permeabilized cells, that allows adding specific substrates, is necessary to investigate the role of each mitochondrial complex (Pesta and Gnaiger, 2012), and to analyze the mitochondrial respiratory profile in different respiratory states (ROUTINE, LEAK, OXPHOS, and ETS), as reported by Gnaiger et al. (2019).

In the present study, we demonstrated that the expression of the mutated protein SOD1(G93A) induces mitochondrial dysfunction, interfering with oxidative phosphorylation mediated by complex I and reducing the coupling efficiency and the mitochondrial membrane potential. Moreover, we provide evidence that ASCs-exosomes are able to revert the mitochondrial dysfunction induced by mutant SOD1(G93A) protein in NSC-34 cells, adding new insights to their neuroprotective action and endorsing the idea that these extracellular vesicles represent a promising strategy for the treatment of ALS.

## Materials and Methods

### Expression Vectors

The plasmids pcDNA3-SOD1(WT) and pcDNA3-SOD1(G93A) were purchased from Addgene (Cambridge, United States) and used as template to amplify by PCR the respective cDNA. Briefly, hSOD1 cDNAs were amplified with AccuPrime DNA polymerase (Invitrogen) and cloned in *Sgf*I and *Mlu*I sites of pCMV6-AN-His-HA plasmid (OriGene) to generate a vector, expressing the human SOD1 gene tagged at the N-terminus with polyhistidine (His) tag and HA. For lentiviruses preparation, the His-HA tagged genes were excised from pCMV6-HIS-HA plasmids and subcloned into the *Bam*HI and *Xho*I sites of the vector pENTR1A (w48-1, Addgene). The resulting vectors were then recombined with pLenti CMV/TO Puro DEST (670-1, Addgene) using Gateway LR-Clonase (Life Technologies) to get the lentiviral vectors expressing hSODWT and its mutant under the control of a doxycycline-inducible promoter. Lentiviruses were then prepared in accordance with the protocols detailed by Campeau et al. (2009), meeting Biosafety Level 2 (BSL-2) requirements.

### Transduction and Generation of Inducible Cell Lines

The murine NSC-34 motoneuron-like cell line was purchased from CELLutions Biosystem Inc. (Ontario, Canada). To establish an inducible cell line, NSC-34 cells were primarily transduced with the pLentiCMV_TetR_Blast vector (Addgene), constitutively expressing the tetracycline (Tet) repressor under the control of a CMV promoter, and selected for 7 days using 10 μg/ml Blasticidin (Sigma-Aldrich). The stable cells were infected with the lentiviral vectors expressing hSOD1(WT) or hSOD1(G93A) genes in the presence of 4 μg/ml polybrene and selected by using 5 μg/ml puromycin. The overexpression of transgenes was induced with 5 μg/ml doxycycline (Clontech) for 24 h in culture.

### Cells Culture

We used NSC-34 naïve cells, NSC-34 cells stably transfected with the human wild type SOD1 gene, NSC-34 SOD1(WT), or the mutated SOD1(G93A) gene, NSC-34 SOD1(G93A). The SOD1 gene expression is under the control of a DOXYcycline-inducible promoter (Bonafede et al., 2016). Cells were cultured using DMEM with 10% FBS, 100 U/ml penicillin and 100 μg/ml streptomycin (all from GIBCO LifeTechnologies, Milan, Italy). The cultures were incubated at 37°C/5% CO_2_. For the experiments, cells (1 × 10^6^) at the 7–9 passages were used. All the experiments were performed in triplicate.

The NSC-34 naïve cells were seeded in the culture medium and after 24 h the cells were harvested, pelleted by centrifugation and re-suspended in warm DMEM.

The NSC-34 SOD1(WT) and NSC-34 SOD1(G93A) cells were seeded and, after 1 day, 5 μg/ml of doxycycline (Doxycycline, Clontech) (DOXY+) was added to the culture medium for 24 h to induce the expression of the human SOD1 genes. In the text, these cells will be called WTDOXY+ and G93ADOXY+. The absence of doxycycline in the culture medium (DOXY−) is used as a negative control (WTDOXY− and G93ADOXY−). After the induction, we harvested the cells, pelleted by centrifugation and re-suspended in 37°C DMEM for intact cells or MIR05 (0.5 mM EGTA, 3 mM MgCl2⋅6H2O, 60 mM K-lactobionate, 20 mM taurine, 10 mM KH2PO4 20 mM Hepes, 110 mM sucrose, and 1 mg/ml bovine serum albumin, pH 7.1 at 30°C) for permeabilized cells.

The adipose-derived stem cells were used to isolate exosomes. Cells were isolated from inguinal adipose tissues of 8/12 week-old C57BL/6 mice (Charles River, Italy), as previously described (Bonafede et al., 2016). Briefly, the inguinal fat was incubated in Hank’s Balanced Salt Solution (HBSS, Life Technologies) with collagenase type I (Life Technologies) and bovine serum albumin (BSA, AppliChem). Centrifugation was performed to obtain a stromal vascular fraction, that was resuspended in NH_4_Cl, centrifuged and filtered through a 40-μm nylon mesh to remove cellular debris. Cells were cultured using DMEM with 10% FBS, 100 U/ml penicillin and100 μg/ml streptomycin. The ASCs cultures were incubated at 37°C/5% CO_2_. Murine ASCs were carachterized by immunophenotype using monoclonal antibodies, as previously described (Bonafede et al., 2016).

### Western Blot of NSC-34 Cells

The NSC-34 naïve, WTDOXY+, G93ADOXY+, and G93ADOXY− cells were lysed for 20 min at 4°C in RIPA buffer supplemented with protease inhibitors (Thermo Fisher Scientific Pierce) and proteins concentration was determined using a Bradford assay (Sigma). Denatured samples, obtained by boiling at 95°C in Laemmli SDS-sample buffer, were separated by 12% SDS-polyacrylamide gel electrophoresis, and electro transferred on nitrocellulose membrane. The membranes were blocked for 1 h at room temperature in TBST buffer (10 mM Tris–HCl, 100 mM NaCl, 0.1% (v/v) Tween 20, pH 7.5) containing 5% milk, followed by overnight incubation at 4°C with primary antibodies: anti-SOD1 (α-SOD) (Novus Biologicals, Littleton, CO, United States, 1:1000), anti-hemoagglutinin (α-HA) (Bethyl Laboratories, 1:1000) to recognize the HA epitope expressed by the human SOD1(G93A) and SOD(WT) lentiviral vector and anti-α-tubulin (Santa Cruz Biotechnology, Santa Cruz, CA, United States, 1:5000) was used as housekeeping gene. The membranes were processed with Pierce ECL Plus from Thermo Fisher Scientific (Rockford, IL, United States).

### ASCs-Exosomes Isolation and NSC-34 Treatment

Exosomes were isolated from the culture medium of 8 × 10^6^ ASCs. The cells were cultured to confluence and, to avoid any contamination of shed membrane fragments and vesicles from serum, 48 h of FBS deprivation was performed. Cell culture supernatants were then collected and PureExo Exosome isolation kit (101Bio, CA, United States) was used for exosomes isolation, following the manufacturer’s protocol. Exosomes were resuspended in 100 μl of PBS and used directly or stored at −80°C. The determination of the protein content of exosomes was performed by Bicinchoninic Protein Assay (BCA method) using the manufacturer’s protocol (Thermo Fisher Scientific^TM^ Pierce^TM^ BCA^TM^ Protein Assay).

The treatment with ASCs-exosomes was performed on G93ADOXY+ cells. The cells were seeded and incubated for 3 h with or without ASCs-exosomes (EXO) at a final concentration of 0.2 μg/ml in the culture medium. Control cells were treated with the same amount of PBS.

### ASCs-Exosomes Characterization: Electron Microscopy and Western Blot Analysis

Exosomes were fixed in 2% glutaraldehyde in DNase/RNase-Free Distilled Water (for 10 min on 150 mesh formvar and carbon-coated copper grids (Società Italiana Chinici, Rome, Italy), and dried under a hood. The images were acquired with a transmission electron microscopy (TEM) using a Morgagni 268D electron microscope (Philips, Andover, MA, United States) operating at 80 kV and equipped with a Megaview II camera (Olympus corporation, Tokyo, Japan) for digital image acquisition.

To perform Western blot of ASCs-exosomes, the proteins were denatured, separated on 4-12% polyacrylamide gels, transferred onto a nitrocellulose membrane and probed with antibodies against Alix (1:50, Santa Cruz Biotechnology, Q-19:sc-49268), heat shock protein 70 (HSP70, 1:100 Santa Cruz Biotechnology, sc-1060), and tetraspanine CD81 (1:100 Santa Cruz Biotechnology, sc-9158). The appropriate HRP-conjugated secondary antibodies against primary antibody (all secondary antibodies from Dako Agilent) were used. ASCs lysates were used as positive control. The blots were then incubated with a chemiluminescent HRP substrate and detected with G:BOX F3 GeneSys (Syngene, United Kingdom).

### High Resolution Respirometry

We used Oxygraph-2K (Oroboros Instruments, Innsbruck) to analyze mitochondrial respiratory capacity (complexes I-IV) in the NSC-34 naïve, WTDOXY+, WTDOXY−, G93ADOXY+, and G93ADOXY− cell lines. In particular, protocols for intact and permeabilized cells were used to evaluate cellular oxygen consumption (Pesta and Gnaiger, 2012). The oxygen respiratory flux was expressed per million cells or flux control ratio (FCR) relative to the maximal uncoupled ETS capacity. Instrumental and chemical background fluxes were opportunely calibrated as a function of oxygen concentration using DatLab software (Oroboros Instruments). The HRR was used to determine the rate of oxygen consumption in the various respiratory states: ROUTINE and LEAK respiration, uncoupled maximal ETS capacity and ROX (residual oxygen consumption). The ROUTINE state respiration is recorded in physiological coupling condition and depends on cellular energy demands, the LEAK respiration is the oxygen consumption associated with dissipative processes compensating for proton and electron leak and cations cycling. The ETS capacity is the maximal oxygen consumption measured at the optimal uncoupler (Carbonyl Cyanide 3-Chlorophenylhydrazone, CCCP) concentration that dissipates the electrochemical proton gradient. After the inhibition of the ETS machinery, oxygen consumption in the ROX state was recorded.

### Intact Cells

Respiration of intact cells was measured applying a coupling control protocol (Pesta and Gnaiger, 2012) After stabilization of ROUTINE respiration with addition of pyruvate and malate (5 mM and 2 mM, respectively; Sigma Aldrich), the ATP-synthase inhibitor oligomycin (Omy, 2.5 μM; Sigma Aldrich) was added to obtain a measure of LEAK respiration, followed by titration of CCCP (0.5 μM steps; Sigma Aldrich) to evaluate the maximum oxygen flux (ETS). Finally, Rotenone (Rot) and Antimycin A (Ama) (2 and 2.5 μM, respectively; Sigma Aldrich) were added to inhibit complex I and III, respectively, in order to obtain ROX.

### Permeabilized Cells

The respiration of permeabilized cells was determined using substrate-uncoupler-inhibitor titration (SUIT) modified protocols (Pesta and Gnaiger, 2012). To investigate the contribution of different mitochondrial complexes (I-IV) to respiratory capacity, cells were permeabilized with the mild detergent digitonin (16 μM, Sigma Aldrich). This concentration was evaluated in preliminary experiments as suitable to achieve full permeabilization of cells allowing the access of substrates and ADP to mitochondria without compromising mitochondrial function. Pyruvate, Malate (PM; 5 mM and 2 mM, respectively) and successively Glutamate (G; 10mM), were used to determine complex I activity (PMG). The ADP (5mM, Sigma Aldrich) was added to stimulate the OXPHOS capacity. To activate the complex II, the succinate was added (S; 10 mM, Sigma Aldrich). The presence of all these substrates (PMGS) allows detecting the respiratory activity of linked complexes I and II. The contribution of complexes III and IV to respiratory activity is always present, although they were not stimulated by the addition of specific substrates.

To analyze the ETS capacity, steps titrations with the uncoupler CCCP (0.5 μM steps; Sigma Aldrich) were performed. Rot and Ama (2 and 2.5 μM, respectively; Sigma Aldrich) were added to inhibit complex I and III determining ROX.

### Mitochondrial Membrane Potential

The mitochondrial transmembrane potential was measured by cytofluorimetry, using the fluorescent dye 3,3′-dihexyloxacarbocyanine iodide [DiOC6(3), Sigma Aldrich]. DiOC6(3) is a green fluorescent membrane dye that has been used to detect mitochondrial membrane potential in live cells. The fluorescence of the dye is enhanced when incorporated into mitochondrial membranes. Changes in mitochondrial membrane potential were assessed in cells by measuring fluorescence intensity due to a conformational change of the dye. The G93ADOXY− cells, G93ADOXY+, and G93ADOXY+ treated with exosomes (1 × 10^6^) were processed as described above. Cells were resuspended in PBS containing DiOC6(3) (1 nM), incubated at 37°C for 15 min, immediately analyzed by flow cytometry with a FACSCanto II (BD) and analyzed with FlowJo software (Treestar Inc.).

### Statistics

Statistical analyses were performed with GraphPad Prism software. Student’s *t*-test, one-way ANOVA (Tukey correction for multiple testing) and two-way ANOVA (Holm-Sidak correction) were used to compare the means. For each experiment, the statistical analysis used is reported in the figure legends. For all analysis, significance differences are indicated as ^∗^*p* < 0.05, ^∗∗^*p* < 0.01, ^∗∗∗^*p* < 0.001, ^∗∗∗∗^*p* < 0.0001. Data are presented as mean with standard deviation.

## Results

### Mitochondrial Bioenergetics of Intact Naïve NSC-34 Cells

**FIGURE 1:**
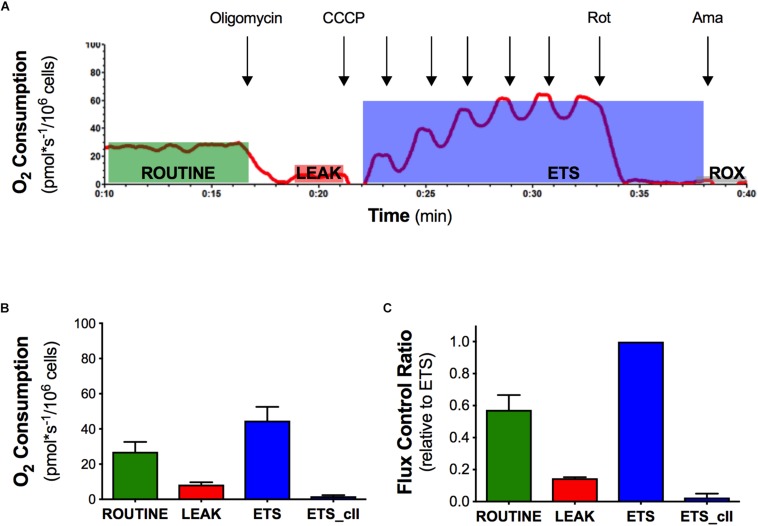
Oxygen consumption and flux control ratios of the naïve NSC-34 intact cells obtained by high resolution respirometry. **(A)** Rate of oxygen consumption of intact naïve NSC-34 measured at 37°C in DMEM in the following states: the ROUTINE respiration sustained by pyruvate, malate, and endogenous substrates; the LEAK respiration obtained following addition of olygomicin; the maximal uncoupled ETS capacity (addition of CCCP) and the ROX, residual oxygen consumption following addition of the inhibitor of complex III antimycin A. The red line represent the rate of oxygen consumption normalized per number of cells. **(B)** Quantitative analysis of data of oxygen consumption in the different respiratory states. **(C)** Flux control ratios, data normalized to the ETS. Data in **(B,C)** are represented as mean ± SD, *n* = 7.

In order to evaluate the effect of the mutant SOD1 protein on mitochondrial function, we analyzed the mitochondrial activity profile in terms of oxygen consumption and flux control ratios in intact naïve NSC-34 cells (Figure 1). We measured ROUTINE, LEAK, ETS, and ETS sustained by complex II ETS(cII) respiratory states in NSC-34 cells, a condition in which mitochondria exploit intracellular available substrates (Figure 1A). The graph in Figure 1B shows data of oxygen consumption recorded in these respiratory states in the cells. The ROUTINE state, that is relative to unstimulated endogenous oxygen consumption, has a value of 27 ± 5.5 pmol^∗^sec^–1^/10^6^cells. The flux control ratios, obtained by normalizing the oxygen consumption data of each respiratory states to the ETS (that represents the maximal uncoupled oxygen consumption), shows an oxygen consumption in the ROUTINE equal to 57%, and 14% in the LEAK respiratory states (Figure 1C). These data characterize the mitochondrial bioenergetics profile of the intact naïve NSC-34 cells, necessary to study the effect of the G93A point mutation in the SOD1 protein.

### Expression of the Human SOD1 Protein in NSC-34 Transfected Cells

**FIGURE 2:**
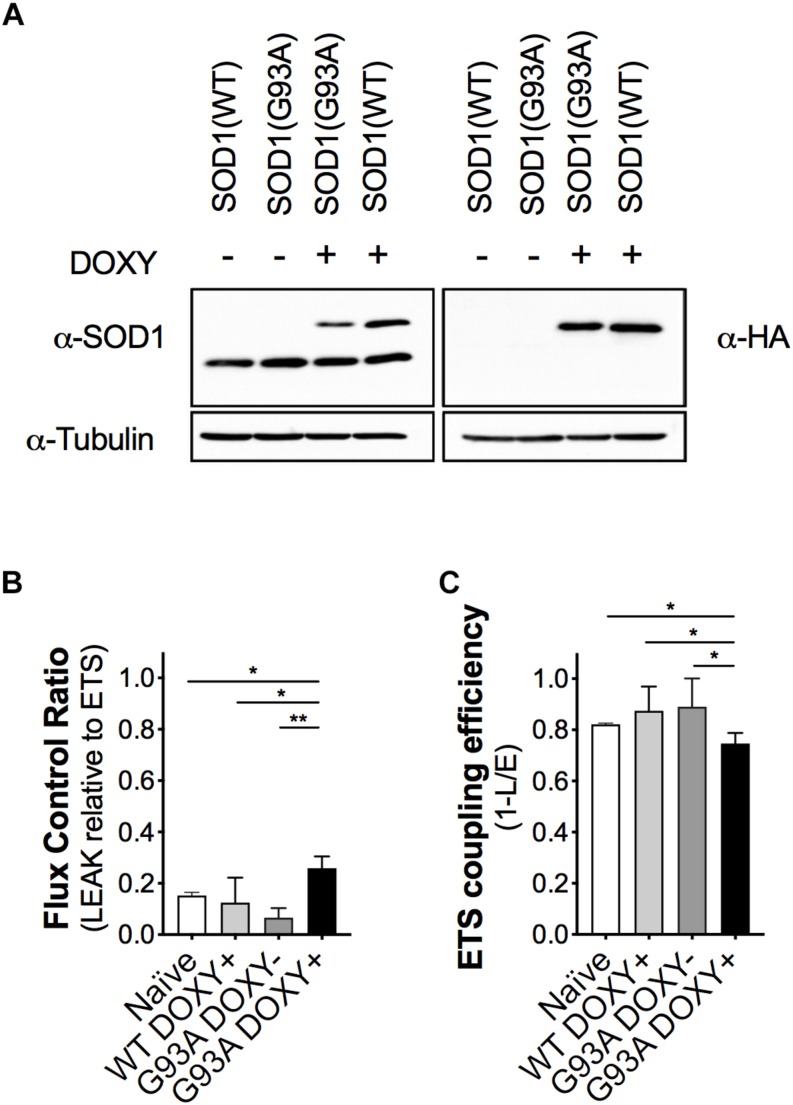
The expression of the human mutant SOD1(G93A) in NSC-34 cells impairs mitochondrial function. **(A)** Representative Western blot assay performed on cells lysates of WTDOXY–, G93ADOXY–, G93ADOXY+, and WTDOXY+ cells. Endogenous mouse SOD1 (mSOD1) immunoreactivity was detected in all samples with anti-SOD1 antibody (left panel), whereas HA-tagged human SOD1 (hSOD1) was detected only in (DOXY+) samples following incubation for 24 h with doxycycline (right panel), *n* = 3. **(B)** Mitochondrial flux control ratios of naïve, WTDOXY+, G93ADOXY–, and G93ADOXY+ intact cells in the LEAK state relative to maximal ETS capacity. **(C)** ETS coupling efficiency of naïve, WTDOXY+, G93ADOXY–, and G93ADOXY+ intact cells determined as 1-(L/E). One-way ANOVA analysis; ^∗^*p* < 0.05, ^∗∗^*p* < 0.01. Data are represented as mean ± SD, *n* = 4.

To evaluate the impact of the mutant SOD1 protein on mitochondrial bioenergetics, we confirmed the expression of the human proteins (WT or G93A) in the NSC-34 cells. As expected, we detected endogenous murine SOD1 protein in all cell types and the human SOD1 protein (with the HA-tag) only in WTDOXY+ and G93ADOXY+ cells (Figure 2A).

### Mitochondrial Bioenergetics of Intact NSC-34 Transfected Cells

We analyzed mitochondrial respiratory capacity (complexes I-IV) on WTDOXY+, WTDOXY−, G93ADOXY+, and G93ADOXY− cells. We found that WTDOXY− show the same mitochondrial bioenergetics profile of WTDOXY+ cells (data not shown). The mitochondrial function of G93ADOXY+ was compared to the G93ADOXY−, WTDOXY+, and naïve cells. The analysis of the flux control ratios reveals significant differences between groups for the LEAK values [*F*(3,12) = 13.29 *p* = 0.0004, *n* = 4]. Indeed G93ADOXY+ cells show a significant increase in the LEAK compared to G93ADOXY− (*p* = 0.001), to WTDOXY+ (*p* = 0.01), and to naïve cells (*p* = 0.03) (Figure 2B). Consistently, the expression of human SOD1 mutated protein significantly affects the ETS coupling efficiency (1-L/E), which is reduced in G93ADOXY+ cells compared to G93ADOXY− (–17%, *p* = 0.02) to WTDOXY+ (–15%, *p* = 0.01), and to naïve cells (–10%, *p* = 0.03) (Figure 2C). LEAK relative to ETS and ETS coupling efficiency did not show other significant differences between the naïve, WTDOXY+ and G93ADOXY− cells (Figures 2B,C). The results demonstrate that the expression of mutated human SOD1 protein impairs metabolic efficiency in mitochondria of intact cells.

The FCR in the LEAK and the ETS coupling efficiency of WTDOXY+ cells are not significantly different compared to naïve cells, indicating that the induction with doxycycline does not affect the mitochondria. Moreover, the expression of human WT SOD1 protein is not responsible for the mitochondrial bioenergetics alteration (Figure 2). These data demonstrate that the mitochondrial dysfunction is related to the SOD1(G93A) expression.

### Mitochondrial Bioenergetics of Permeabilized NSC-34 SOD1(G93A) Cells

**FIGURE 3:**
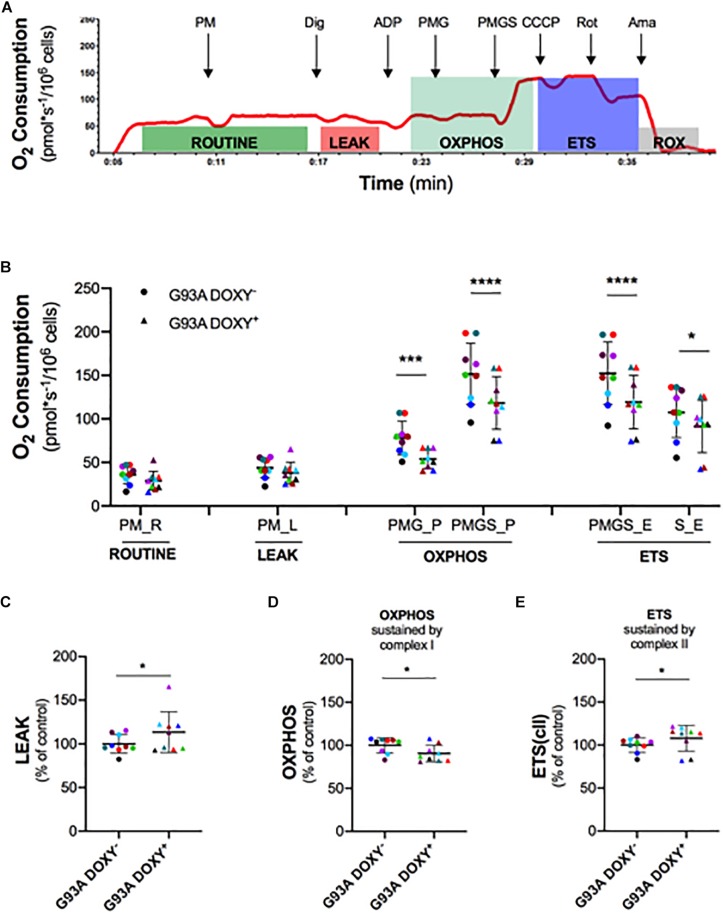
Mitochondrial complexes contribution to oxidative phosphorylation in permeabilized G93ADOXY+ and G93ADOXY– cells. **(A)** Rate of oxygen consumption (red line) of permeabilized G93ADOXY– cells measured at 37°C in Mir05 in the following states: the ROUTINE respiration sustained by pyruvate and malate (PM) and endogenous substrates; the LEAK respiration evaluated following permeabilization with digitonin; the ADP addition stimulated OXHPOS capacity sustained by complex I (pyruvate, malate and glutamate, PMG) and complex II (pyruvate, malate, glutamate and succinate, PMGS); the maximal uncoupled ETS capacity (addition of CCCP); ETS sustained by complex II following inhibition of complex I by rotenone; the ROX, residual oxygen consumption, following addition of the inhibitor of complex III antimycin A. **(B–E)** Dot plots corresponding to respirometry experiments, showing data from all samples. Each dot corresponds to one sample. The line shows the mean with standard deviation. Paired samples are represented by symbols of the same color. **(B)** Quantitative analysis of the rate of oxygen consumption of G93ADOXY– and G93ADOXY+ permeabilized cells: the values relative to ROUTINE, LEAK, OXPHOS, and ETS for paired samples are reported. **(C–E)** Flux control ratios, normalized to the maximal uncoupled ETS capacity, of G93ADOXY–, and G93ADOXY+ permeabilized cells in the LEAK, OXPHOS and ETS(cII) respiratory states. Data are represented as percentage of control (G93ADOXY– cells). Data of flux control ratios (mean ± SD) in G93ADOXY– cells are: **(C)** LEAK (0.28 ± 0.03), **(D)** OXPHOS(PMG) (0.50 ± 0.04), and **(E)** ETS(cII) (0.69 ± 0.05). Analysis of G93ADOXY– vs. G93ADOXY+ cells at various respiratory states was performed using two-way ANOVA (Holm-sidak’s post hoc correction) ^∗^*p* < 0.05, ^∗∗∗^*p* < 0.001, ^∗∗∗∗^*p* < 0.0001 **(B)**; paired student T test **(C–E)** ^∗^*p* < 0.05. *n* = 9.

To investigate the role of SOD1(G93A) in the alteration of mitochondrial complexes, we permeabilized the plasma membrane allowing specific substrates and inhibitor molecules to enter in the cells. The Figure 3A shows a representative rate of oxygen consumption in the respiratory states (ROUTINE, LEAK, OXPHOS, and ETS) analyzed in G93ADOXY− cells. The digitonin, that allows to permeabilize the cells, was added at the end of ROUTINE state. The addition of pyruvate, malate (PM), and glutamate (PMG) stimulates the complex I and allow to evaluate its function. The ADP was added at the end of the LEAK state to stimulate the oxidative phosphorylation (OXPHOS). The further addition of succinate (PMGS) stimulates also complex II to assess the synergistic contribution of complex I and complex II. The CCCP was added to induce the ETS state, the rotenone inhibiting the complex I allows to measure the contribution of complex II. Finally, the evaluation of ROX was obtained by adding the inhibitor of complex III antimycin A (Figure 3A).

The comparison of oxygen consumption levels between G93ADOXY− and G93ADOXY+ cells in the analyzed respiratory states showed significant differences [*F*(1,48) = 78.77 *p* < 0.0001, *n* = 9]. The oxygen consumption of complex I (PMG) in the OXPHOS is significantly reduced in G93ADOXY+ cells compared to G93ADOXY− (–27%, *p* < 0.0004). In G93ADOXY+ cells, the oxygen consumption remains at lower levels compared to G93ADOXY− (–22%, *p* < 0.0001) even after stimulation of complex II (PMGS) (Figure 3B). In the OXPHOS, the relative contribution of the complex I and complex II in G93ADOXY− is about 50% each, while in G93ADOXY+ cells the contribute is 45% and 55%, respectively.

Moreover, the oxygen consumption of G93ADOXY+ cells in the ETS state (PMGS) was reduced (–22%, *p* < 0.0001), compared to G93ADOXY−. The contribution of the complex II in the ETS (S) is significantly decreased (–15%, *p* = 0.01) compared to G93ADOXY− (Figure 3B). These data suggest that the oxygen consumption linked to OXPHOS and ETS respiratory states is significantly reduced in G93ADOXY+ cells.

To evaluate the relative contribution of the various complexes to mitochondrial respiration we analyzed the flux control ratios (Figures 3C–E and Supplementary Table S1). The G93ADOXY+ cells showed an increase in the LEAK compared to G93ADOXY− (+14%, *p* = 0.04, Figure 3C), indicating that ETS is dissipative and highlighting a possible alteration of the quality of inner mitochondrial membrane. Moreover, in G93ADOXY+ cells, a decrease of the flux control ratio in the OXPHOS sustained by complex I (PMG) was detected compared to G93ADOXY− (–10%, *p* = 0.03, Figure 3D), reinforcing the idea that the complex I is mainly affected. The contribution of complex II to ETS capacity is increased in G93ADOXY+ cells compared to G93ADOXY− (+7%, *p* = 0.04, Figure 3E), suggesting a compensatory activity of complex II.

Altogether, these data confirm that the human SOD1 mutant protein is involved in mitochondrial dysfunction and demonstrate that the mutated protein affects the coupling efficiency and impairs the functionality of complex I.

### Isolation and Characterization of ASCs-Exosomes

**FIGURE 4:**
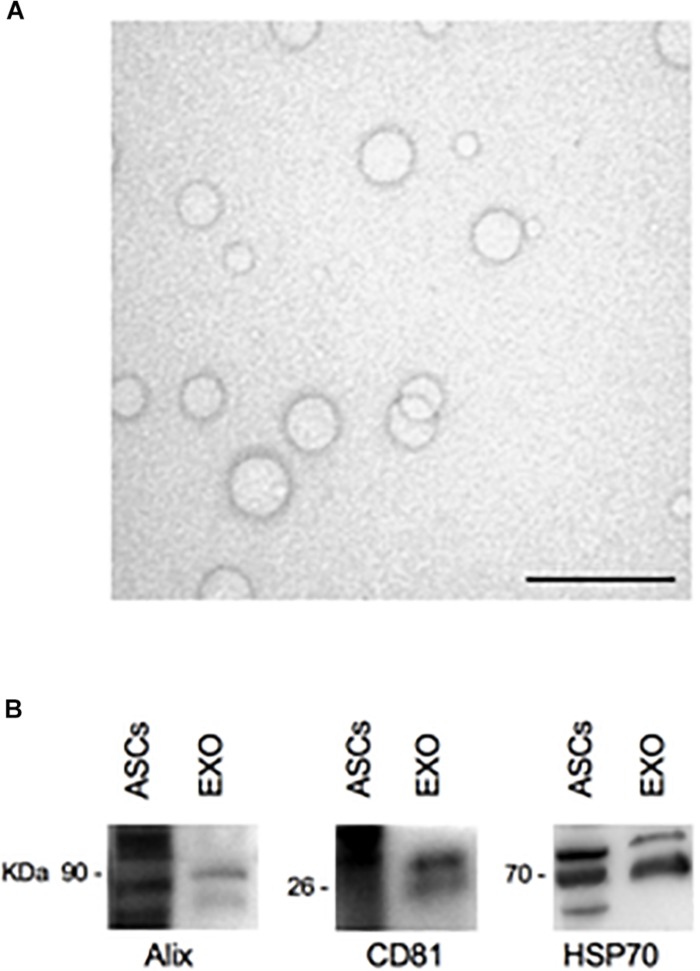
TEM and Western blot analysis of ASCs-exosomes. **(A)** Electron microscopy shows vesicles with characteristic morphology and size of exosomes. Scale bar, 200 nm. **(B)** The blots show Western blot detection of the expression of Alix (90 kDa), CD81 (26 kDa), and HSP70 (70 kDa) in exosomes (EXO); ASCs lysates were used as positive control.

After ASCs-exosomes isolation, their protein concentration was quantified. The yield for each isolation was about 200 μg/ml of protein. Ultrastructural analysis of the exosomes sample by TEM shows the extracellular vesicles with the lipid bilayer, with a diameter ranging from 30 to 120 nm (Figure 4A). Western blot analysis reveals specific exosomal markers of the vesicles, as Alix (90 kDa), HSP70 (70 kDa), and CD81 (26 kDa) (Figure 4B). These results (size, morphology and specific markers) demonstrate that the isolated extracellular vesicles are ASCs-exosomes.

### Exosomes Rescue Mitochondrial Function in NSC-34 SOD1(G93A) Cells

**FIGURE 5:**
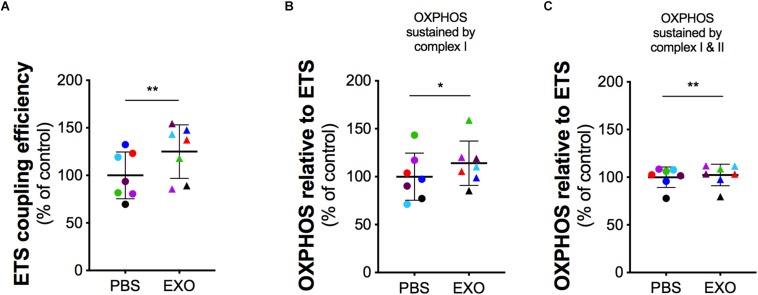
Mitochondrial function in G93ADOXY+ cells after exosomes treatment. Dot plots of data obtained from respirometry experiments, showing the effects of vehicle (PBS), or exosomes (EXO) treatment in DOXY+ cells for all samples. Each dot corresponds to one sample. The line shows the mean with standard deviation. Paired samples are represented by symbols of the same color. **(A)** ETS coupling efficiency in G93ADOXY+ intact cells following treatment with exosomes (EXO) or PBS. **(B)** Flux control ratio of the OXPHOS sustained by complex I (PMG) in G93ADOXY+ permeabilized cells following incubation with exosomes (EXO) or PBS. **(C)** Flux control ratio of the OXPHOS sustained by complex II (PMGS) in G93ADOXY+ permeabilized cells following incubation with exosomes (EXO) or PBS. Comparison of EXO vs. PBS treated G93ADOXY+ cells was performed using paired *t*-test. Data are represented as percentage of control (PBS). Data of flux control ratios (mean ± SD) in PBS treated cells cells are: **(A)** ETS coupling efficiency (0.54 ± 0.13), **(B)** OXPHOS(PMG) (0.29 ± 0.07), and **(C)** OXPHOS(PMGS) (0.81 ± 0.12). Paired *T* test ^∗^*p* < 0.05, ^∗∗^*p* < 0.01. *n* = 7.

In order to assess the potential rescue effect of ASCs-exosomes on mitochondrial dysfunction in G93ADOXY+, these cells were treated with ASCs-exosomes or vehicle (PBS) (Figure 5 and Supplementary Table S2). In intact treated G93ADOXY+ cells, data of ETS coupling efficiency showed an increase of 25% (*p* = 0.005) compared to control (Figure 5A). Furthermore, in permeabilized cells treated with exosomes, we observed a significant increase of 14% (*p* = 0.04) of flux control ratio sustained by complex I (PMG) in the OXPHOS compared to control cells (Figure 5B). This data indicates that ASCs-exosomes treatment leads to a partial recovery of complex I activity. The flux control ratio sustained by both complex I and II (PMGS) in the OXPHOS is significantly increased (*p* = 0.001) by exosomes treatment in permeabilized G93ADOXY+ cells (Figure 5C). Together, these data indicate that the incubation with ASCs-exosomes produced an evident restoration of mitochondrial function detected by HRR.

**FIGURE 6:**
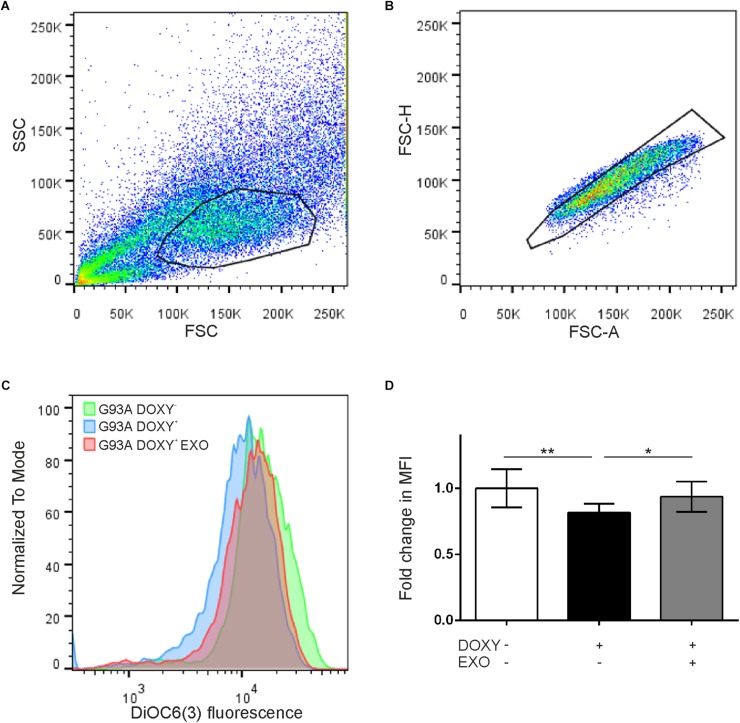
Flow cytometry for mitochondrial membrane potential evaluation in G93ADOXY+ and G93ADOXY– cells. **(A,B)** Representative dot plots of flow cytometry of all events collected using gates to exclude dead cells and debris **(A)** and to select the singlets (single cells) **(B)**. Cell size (forward scatter, FSC) vs. cellular granularity (side scatter, SSC) data are plotted. **(C)** The histograms show the intensity of DiOC6(3) probe in the G93ADOXY– cells, G93ADOXY+ cells, and G93ADOXY+ cells treated with exosomes (EXO). **(D)** Measurement of mitochondrial membrane potential with DiOC6(3) probe in cells G93ADOXY– and G93ADOXY+ following treatment with exosomes. Data are reported as median fluorescence intensity (MFI) of the probe relative to the G93ADOXY– cells. The analysis was performed using one-way ANOVA with Tukey correction; *p* < 0.05. Data are represented as mean ± SD, ^∗^*p* < 0.05, ^∗∗^*p* < 0.01. *n* = 9.

In addition, to evaluate if the SOD1(G93A) protein affects the mitochondrial membrane potential, we performed the cytofluorimetry using the DiOC6(3) probe, a dye that is transported into the mitochondria by the negative mitochondrial membrane potential and thus concentrates within the mitochondrial matrix (Figures 6A,B). An alteration of the mitochondrial membrane potential implies a reduction in the dye fluorescence intensity (Figure 6C). Data obtained by cytofluorimetry showed significant changes between groups [*F*(1.684, 13.47) = 9.058 *p* = 0.0043, *n* = 9] that the median fluorescence intensity in G93ADOXY+ cells is reduced by 20% (*p* = 0.007) compared to G93ADOXY− control cells (Figure 6D), indicating that the G93A mutation interferes with the mitochondrial membrane potential and supporting the reduction in ETS coupling efficiency reported above. Consistently, the ASCs-exosomes treatment of G93ADOXY+ restored the mitochondrial membrane potential to values similar to the control condition (G93ADOXY−), with an increase of 16% of median fluorescence intensity (*p* = 0.02) compared to the untreated G93ADOXY+ cells (Figure 6D).

These results highlight that ASCs-exosomes treatment rescues the mitochondrial dysfunction and the mitochondrial membrane potential, demonstrating a possible therapeutic use of these vesicles in diseases featured by mitochondrial alterations, as ALS.

## Discussion

In amyotrophic lateral sclerosis, mitochondrial dysfunction has been proposed to play a key role in the selective vulnerability of motoneurons (Cozzolino and Carrì, 2012). It has been reported histopathological and functional mitochondrial alterations in both ALS murine models and patients (Jung et al., 2002; Dupuis et al., 2003; Higgins et al., 2003; Sasaki and Iwata, 2007).

It has been demonstrated that misfolded mutated SOD1 proteins aggregate, leading to an imbalance between ROS production and removal, causing net ROS accumulation (Golpich et al., 2017). The increase of oxidative stress damages lipids, proteins and carbohydrates of mitochondrial membranes (Golpich et al., 2017). Indeed, morphological analysis of mitochondria of NSC-34 SOD1(G93A) revealed altered membranes, characterized by abnormal cristae (Raimondi et al., 2006). Alterations of mitochondria, associated with reduced mitochondrial membrane potential, have been reported also in mutated human SOD1(G37R) neuroblastoma cells and in SOD1(G93A) primary motoneurons (Kruman et al., 1999; Coussee et al., 2011). A recent meta-analysis shows that in SOD1 transgenic mice the level of ATP production is reduced from the pre-clinical stage (Irvin et al., 2015).

Concerning the study on alterations of mitochondria functionality, the activity of respiratory complexes was evaluated in isolated enzymes or mitochondrial preparations obtained from bioptic samples or cell culture (Menzies et al., 2002; Ghiasi et al., 2012). The main limitation in using isolated mitochondria consists of the requirement of a large amount of material (tissue or cells) and in the risk of damaging the organelle during the isolation procedure. On the other hand, the approach based on respirometry in living cells allows to avoid these limitations and obtain detailed information about mitochondrial impairment of the cellular bioenergetic state (Djafarzadeh and Jakob, 2017a, b).

In fibroblasts obtained from ALS patients, mitochondrial oxygen consumption was found to be reduced in cells bearing the mutated SOD1(I113T) protein (Allen et al., 2014). Another study reported that mitochondrial coupled respiration and coupling efficiency was reduced in intact NSC-34 cells expressing the G93A mutation (Richardson et al., 2013). However, these studies were performed on intact cells, without the possibility to clarify the contribution of single complex of the ETS to aerobic respiration. The mitochondrial dysfunction was also reported in NSC-34 cell line transfected with hTDP-43 gene. Lu et al. (2012) reported that the presence of mutated TDP-43 protein in transfected NSC-34 cells causes morphological abnormalities, reduces complex I activity and determines a loss of transmembrane potential. In particular NSC-34 cells, following transfection with the mutated protein of interest, have been used to study the pathogenic mechanisms of ALS, including mitochondrial dysfunction (Muyderman et al., 2009).

In the present study, we described the mitochondrial function and the contribution of single complexes to aerobic respiration and mitochondrial coupling efficiency through high-resolution respirometry, that allows measuring mitochondrial respiratory capacity, the specific role of complexes I-IV and their synergic action. With this approach, we preserve mitochondrial integrity, and energy metabolism is evaluated in real time directly on living cells. Moreover, the possible alteration of the mitochondrial membrane potential was studied.

To this purpose, we used an *in vitro* model of ALS disease [NSC-34 SOD1(G93A)]. We characterized the bioenergetic profile of living NSC-34 naïve cells and the impairment of the mitochondrial complexes associated with the mutated SOD1 protein using the HRR. We observed an increase of the dissipative LEAK respiration in intact G93ADOXY+ cells, accompanied by a reduction of the coupling efficiency, compared to the other conditions. These results suggest that the expression of the mutated SOD1 protein in these cells leads to an alteration of mitochondrial membrane integrity. In relation to this, results obtained with DiOC6(3) probe showed that the SOD1(G93A) mutation affects the mitochondrial membrane potential of NSC-34 SOD1(G93A) cells. This observation is in line with Richardson et al. (2013), that found a reduced coupling efficiency and a decreased mitochondrial membrane potential in intact NSC-34 SOD1(G93A) cells using the Seahorse instrument and theTMRM probe.

In our study, we have investigated the contribution of single mitochondrial complexes in the OXPHOS and ETS respiratory states in permeabilized NSC-34 SOD1(G93A) cells, to identify which of them are affected by SOD1(G93A) protein. We demonstrated that the mutant SOD1 protein induces an impairment of the complex I-linked oxidative phosphorylation. Furthermore, in the ETS, following complex I inhibition, we observed an increased activity indicating a compensatory enzymatic action of complex II. The reduction of complex I activity may be due to mitochondrial damage directly carried by mutant SOD1 protein or to an increase of free radical species that reacts with complex I subunits (Yim et al., 1996; Magrane et al., 2009; Murphy, 2009; Abeti et al., 2016).

The observed effect of SOD1(G93A) could be due to the ability of the misfolded mutated protein to bind the VDAC1 (voltage-dependent anion channel 1), blocking the metabolite flux across the outer mitochondrial membrane and reducing the conductance of the channel. This phenomenon results in reduced energy production and in an increase of oxidative stress, contributing to mitochondrial dysfunction (Israelson et al., 2010). It has been observed that the administration of an antioxidant compound in a murine model of ALS counteracts the disease progression and prolongs their lifespan (Miquel et al., 2014). This observation suggests that the mitochondria could represent a crucial target to cure ALS.

In addition, in SOD1(G93A) mice, a neuroprotective effect was found following treatment with ASCs, suggesting that the beneficial effect of the cells is mediated by extracellular vesicles through a paracrine mechanism (Marconi et al., 2013). This leads to the idea that these vesicles are able to mimic the effect of parental cells suppressing the pathological processes involved in many neurodegenerative diseases (Biancone et al., 2012; Bonafede and Mariotti, 2017). In light of these results, we have demonstrated a neuroprotective effect of ASCs-exosomes in NSC-34 cell line carrying ALS mutations, inhibiting cell death (Bonafede et al., 2016). Moreover, exosomes released from mesenchymal stem cells induced a neuroprotective effect on chronic experimental autoimmune encephalomyelitis model (Farinazzo et al., 2018) and on ischemic spinal cord by the improvement of motor function and survival of motoneuron (Zhao et al., 2019). This study reports that after exosomes treatment there is an increase of superoxide dismutase activity in the spinal cord (Zhao et al., 2019). In addition, exosomes derived from modified ASCs were reported to be able to regulate the autophagy and apoptosis ameliorating brain ischemia (Huang et al., 2018).

In order to restore mitochondrial functionality in NSC-34 mutated cells, we used ASCs-exosomes. Our results show that the ASCs-exosomes are able to restore mitochondrial function, in terms of complex I contribution to ETS and the coupling efficiency. Furthermore, exosomes treatment was able to restore the mitochondrial membrane potential. One might wonder if the exosomes are rescuing a detrimental phenotype through a specific effect or if they act generically boosting mitochondrial function. Although this is beyond the goal of our study, the results point out a specific effect of exosomes on impaired OXPHOS sustained by complex I and on ETS coupling efficiency in NSC-34 SOD1(G93A). Nevertheless, we believe that the effect of exosomes on other cells, such as intact naïve NSC-34 cells, could still lead to an increased OXOPHOS sustained by complex I but probably not in an improved ETS coupling efficiency since the cells are not compromised by increased oxidative stress.

Furthermore it could be interesting to compare the effects of exosomes isolated from different sources on mitochondrial function in this *in vitro* ALS model, also to identify the peculiar mechanisms involved. Indeed we can’t exclude that also other exosomes from different sources may affect mitochondrial function. It has been reported that adipose-derived stem cells exosomes and bone marrow-derived exosomes, both of mesenchymal origin, improved mitochondrial function in different *in vitro* models (Lee et al., 2016; Hogan et al., 2019). However further studies are needed to understand the mechanisms by which ACSs-exosomes exert their beneficial and specific role. The presence of SOD1 protein in ASCs-exosomes could result to be particularly interesting in ALS disease, and in particular concerning the mitochondrial alterations related to the disease. Indeed, some studies indicate that forms of misfolded SOD1, deposited on the cytoplasmic surface of the outer membrane of mitochondria in spinal cord motoneurons, reduce the import of mitochondrial proteins and complex I activity (Vande Velde et al., 2008; Israelson et al., 2010). In the line of this, the presence of the SOD1 in exosomes could counteract the mutated SOD1 protein, reducing the mitochondrial dysfunction.

Altogether these data indicate that ASCs-exosomes could represent a valuable approach to target mitochondrial dysfunction in ALS, as well as in other pathologies sharing common mitochondrial impairment.

## Data Availability Statement

The data that support the findings of this study are available from the corresponding authors on request.

## Author Contributions

EC, IS, and RM planned the majority of the experiments and wrote the manuscript. EC, IS, RB, and DP executed most of the experiments. VP performed the Western blot analyses. LS helped in exosomes preparation. FS and CC contributed to the final editing of the manuscript. All authors contributed to the manuscript revision, and read and approved the submitted version.

## Conflict of Interest

The authors declare that the research was conducted in the absence of any commercial or financial relationships that could be construed as a potential conflict of interest.
