# Supplementary material for: ASCs-Exosomes Recover Coupling Efficiency and Mitochondrial Membrane Potential in an in vitro Model of ALS
Source: Front Neurosci. 2019 Oct 17;13:1070. doi: 10.3389/fnins.2019.01070 (PMC6811497; doi:10.3389/fnins.2019.01070)
Supplement: Supplementary file 1 [file Table_1.DOCX]

**Table S1** - Summary of the values of flux control ratios (FCR) in the LEAK, OXPHOS and ETS respiratory states in G93A DOXY^-^ and G93A DOXY^+^ cells.

Data are reported as mean ± standard deviation. (n=9)

|  | **G93A DOXY^-^** | G93A DOXY^+^ | P value  (paired T test) |
| --- | --- | --- | --- |
|  |  |  |  |
| LEAK | 0.28±0.03 | 0.32±0.06 | 0.04 |
| OXPHOS sustained by complex I | 0.50±0.04 | 0.46±0.04 | 0.01 |
| ETS sustained by complex II | 0.69± 0.05 | 0.74± 0.10 | 0.04 |

**Table S2** - Summary of the values of flux control ratios (FCR) in the LEAK and OXPHOS respiratory states in G93A DOXY^+^ cells treated with PBS or ASCs exosomes (EXO). Data are reported as mean ± standard deviation. (n=7)

|  | PBS | EXO | P value  (paired T test) |
| --- | --- | --- | --- |
|  |  |  |  |
| 1-(L/E) | 0.54±0.13 | 0.67±0.15 | 0.005 |
| OXPHOS sustained by complex I | 0.29±0.07 | 0.33±0.07 | 0.04 |
| OXPHOS sustained by complex I & II | 0.81± 0.12 | 0.84± 0.11 | 0.001 |
